# Supplementary material for: Proteogenomics of clear cell renal cell carcinoma response to tyrosine kinase inhibitor
Source: Nat Commun. 2023 Jul 17;14:4274. doi: 10.1038/s41467-023-39981-6 (PMC10352361; doi:10.1038/s41467-023-39981-6)
Supplement: Supplementary file 1 — Supplementary Information file [file 41467_2023_39981_MOESM1_ESM.pdf]

# Supplementary Information

## Proteogenomics of Clear Cell Renal Cell Carcinoma Response to Tyrosine Kinase Inhibitor

Hailiang Zhang, Lin Bai, Xin-Qiang Wu, Xi Tian, Jinwen Feng, Xiaohui Wu, Guo-Hai Shi, Xiaoru Pei, Jiacheng Lyu, Guojian Yang, Yang Liu, Wenhao Xu, Aihetaimujiang Anwaier, Yu Zhu, Da-Long Cao, Fujiang Xu, Yue Wang, Hua-Lei Gan, Meng-Hong Sun, Jian-Yuan Zhao, Yuanyuan Qu, Dingwei Ye, Chen Ding

# Supplementary Figure 1

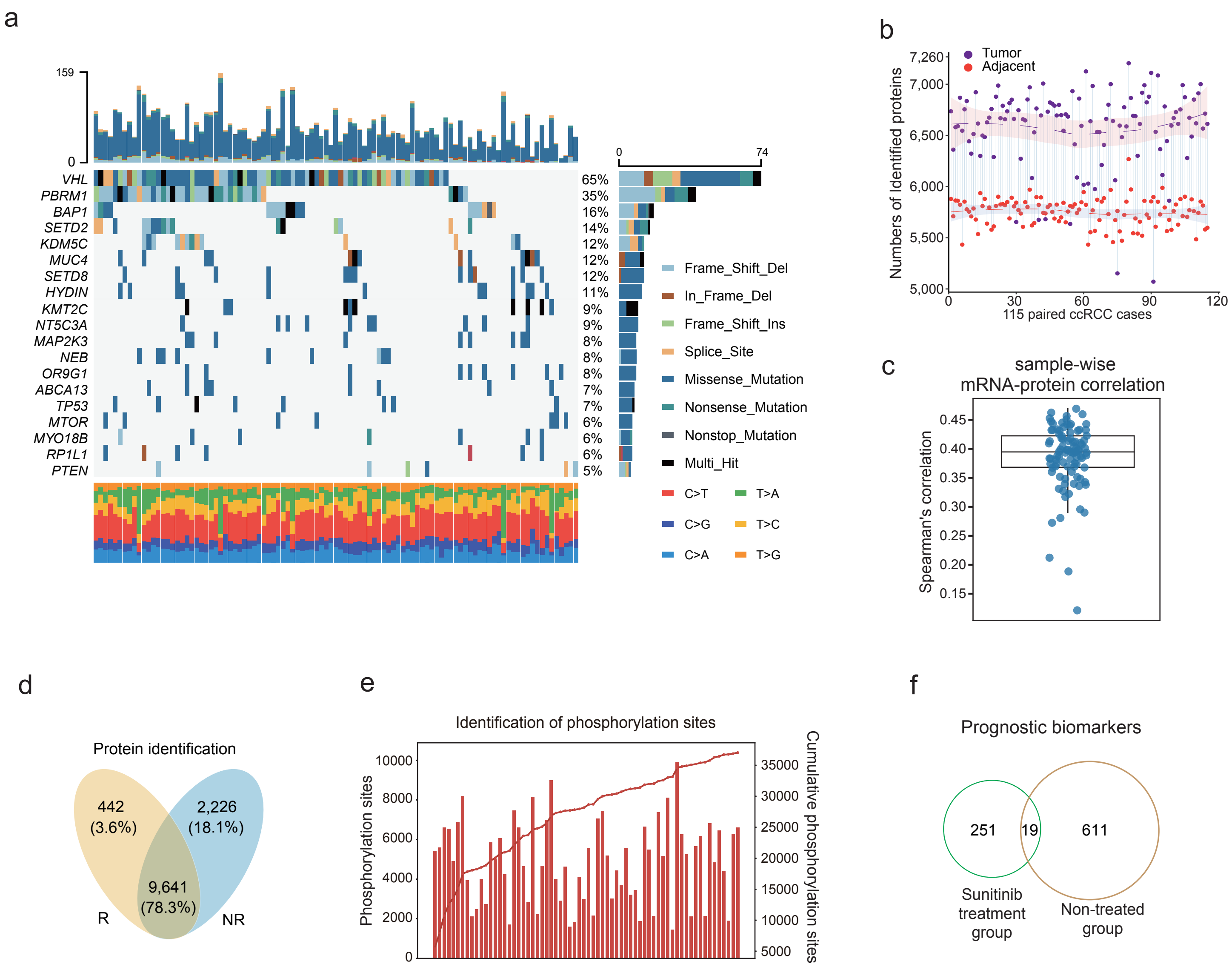

**Supplementary Figure 1. Proteogenomic Analysis of ccRCC Response to Sunitinib.** **a**, Genomic profile of 113 patients with ccRCC who received Sunitinib therapy. **b**, Overview of proteomic profiles for paired ccRCC samples (analyzed patients: $n = 115$). Pairwise comparison of proteins identified in 115 patients. The dashed curves fitted by lasso regression show the distribution of protein identification. The shading that underlies the lasso curves denotes the 95% confidence intervals. **c**, Boxplot showing sample-wise mRNA-protein correlation (analyzed patients: $n = 94$). Boxplots show the median (central line), the 25-75% interquartile range (IQR) (box limits), the ±1.5×IQR (whiskers). **d**, The Venn diagram showing the overlap of proteins identified in Responders and Non-Responders. **e**, Overview of identified phosphosites of 66 ccRCC tumor samples in this study. **f**. Venn plot of prognostic significance of protein expression levels in Sunitinib treatment group and non-treated group. Source data are provided as a Source data file.

# Supplementary Figure 2

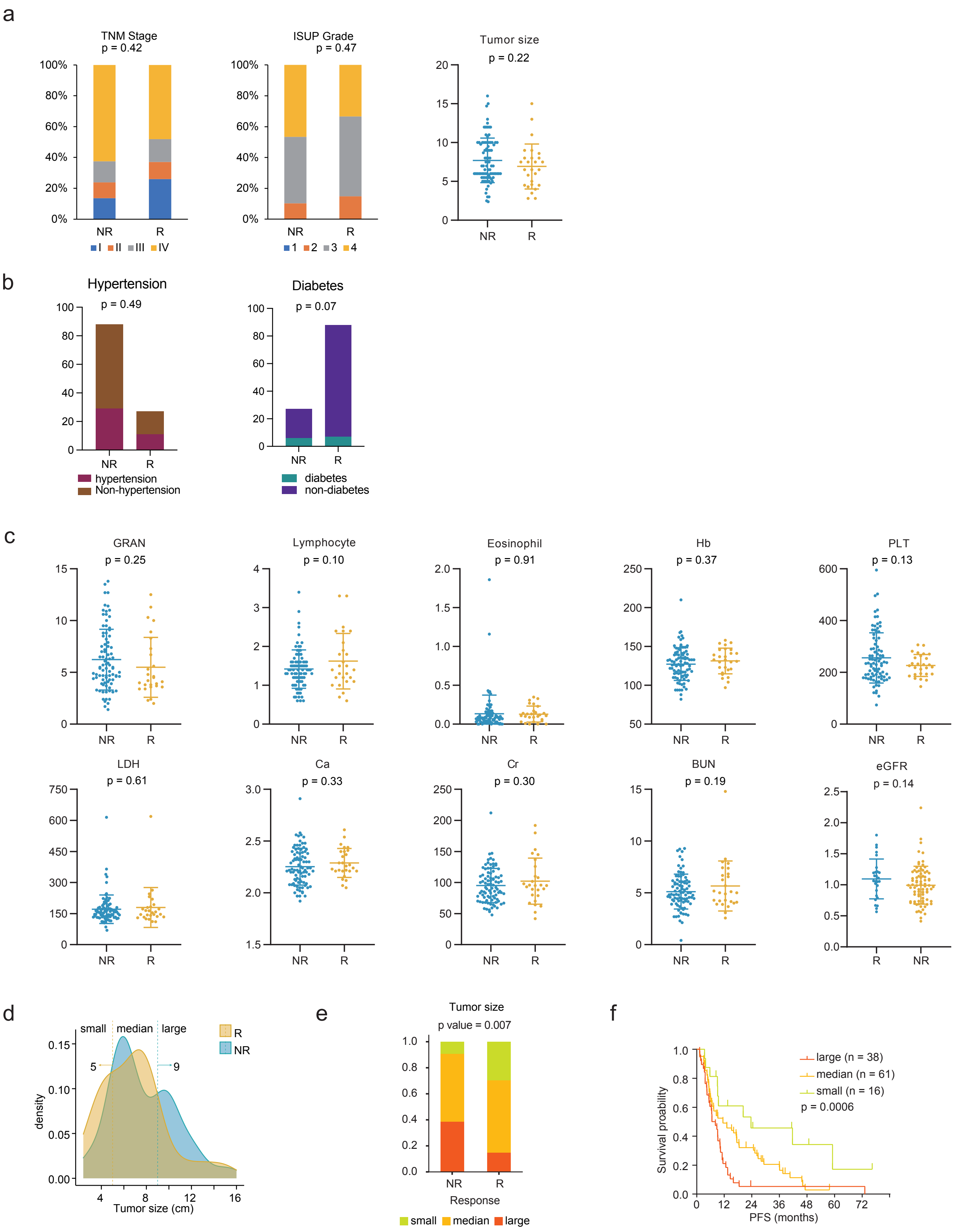

**Supplementary Figure 2. The Clinical Features Associated with Sunitinib Treatment Outcomes.**

**a**, The comparisons of clinical parameters between Responder group and Non-Responder group for the Sunitinib treatment, including TNM stage (at the time of surgery; R, *n* = 27; NR, *n* = 88; Fisher's exact test), ISUP grade (R, *n* = 27; NR, *n* = 88; Fisher's exact test), tumor size (R, *n* = 27; NR, *n* = 88; data are presented as mean values +/- SD, two-sided Student's t test). **b**, The comparisons of clinical parameters between Responder group and Non-Responder group for the Sunitinib treatment, including hypertension (R, *n* = 27; NR, *n* = 88; two-sided Fisher's exact test), diabetes (R, *n* = 27; NR, *n* = 88; two-sided Fisher's exact test). **c**, The comparisons of clinical parameters between Responder group and Non-Responder group for the Sunitinib treatment, including GRAN (R, *n* = 27; NR, *n* = 88), Lymphocyte count (R, *n* = 27; NR, *n* = 88), Eosinophil count (R, *n* = 24; NR, *n* = 81), Hb level (R, *n* = 27; NR, *n* = 88), platelet (PLT) count (R, *n* = 27; NR, *n* = 88), LDH level (R, *n* = 27; NR, *n* = 88), Calcium level (R, *n* = 27; NR, *n* = 88), Creatinine level (R, *n* = 27; NR, *n* = 88), blood urea nitrogen (BUN) level (R, *n* = 27; NR, *n* = 88), estimated glomerular filtration rate (eGFR) level (R, *n* = 27; NR, *n* = 81). Data are presented as mean values +/- SD. P value is derived from two-sided Student's t test. **d**-**e**, Tumor sizes distributions of Responders and Non-Responders (two-sided Fisher's exact test). **f**, Kaplan–Meier curves of progression-free survival (PFS) for patients with different tumor size. Source data are provided as a Source data file.

# Supplementary Figure 3

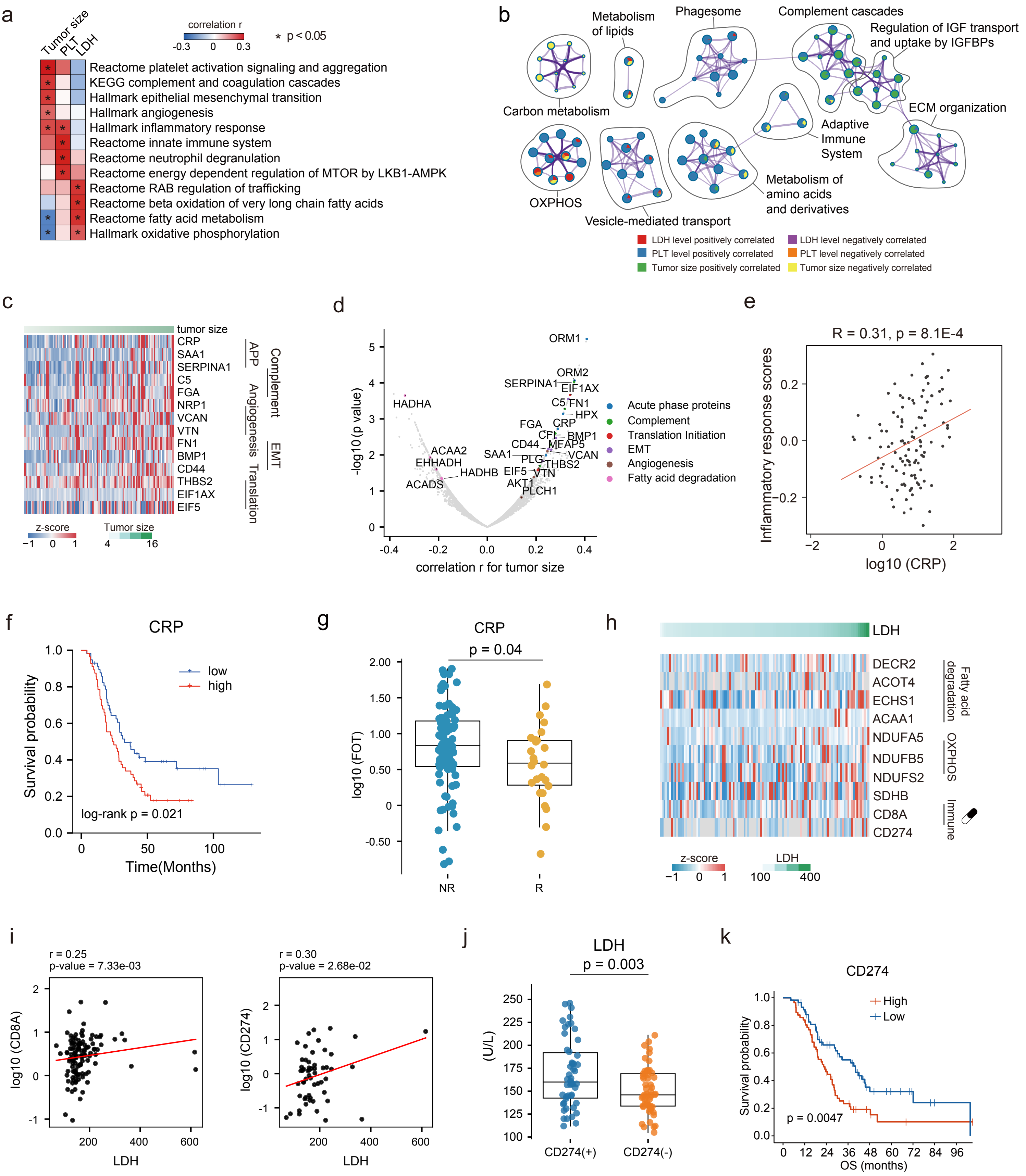

**Supplementary Figure 3. The associations between the Tumor size, plasma PLT and LDH level under the Sunitinib treatment.**

**a**, Tumor size, plasma PLT and LDH level-correlated pathways. Two-sided Spearman's correlation, $*p < 0.05$. **b**, Metascape diagram of proteins correlated with tumor size, PLT and LDH levels. **c**, Heatmap of proteins correlated with the tumor size. **d**, Proteins correlated with tumor size. Proteins were distinguished by the pathways they were involved in. Two-sided Spearman's correlation, $p < 0.05$. **e**, The correlation of CRP abundance and Inflammatory response scores (two-sided Spearman's correlation test). **f**, The Kaplan-Meier curves of OS for patients with different CRP abundances (log-rank test). **g,** CRP abundances in Responders ($n = 26$) and Non-Responders ($n = 87$) (two-sided Wilcoxon rank-sum test). Boxplots show the median (central line), the 25–75% interquartile range (IQR) (box limits), the ±1.5×IQR (whiskers). **h**, Heatmap of proteins correlated with the plasma LDH level. **i**, The correlations of LDH level and CD8A/CD274 abundances (two-sided Spearman's correlation test). **j**, Comparison of plasma LDH levels between PD-L1 positive ($n = 22$) and negative ($n = 50$) ccRCC patients (two-sided Wilcoxon rank-sum test). Boxplots show the median (central line), the 25–75% interquartile range (IQR) (box limits), the ±1.5×IQR (whiskers). **k**, Kaplan-Meier curves of OS for patients with different CD274 (PD-L1) abundances (log-rank test). Source data are provided as a Source data file.

# Supplementary Figure 4

a

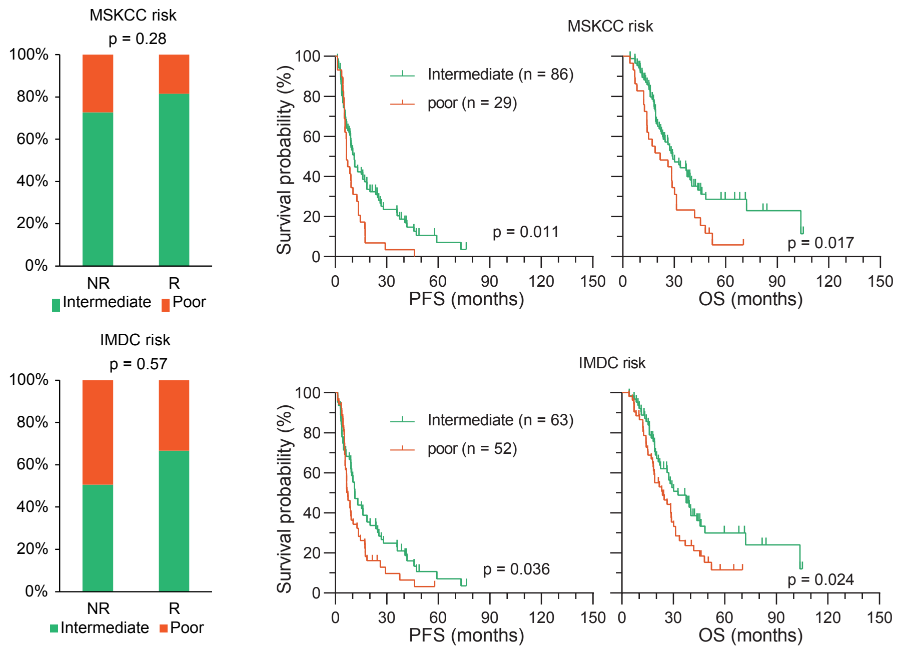


b

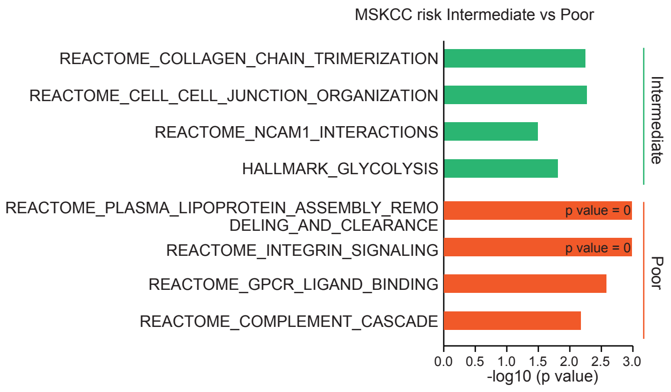


c

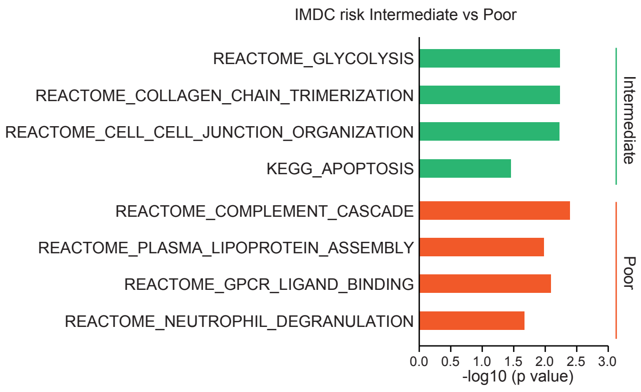


d

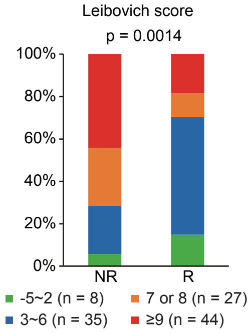


e

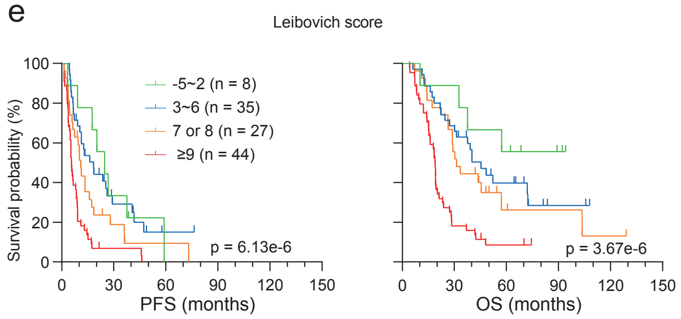

**Supplementary Figure 4. MSKCC and IMDC risk models to prognosticate in advanced RCC.**

**a,** Comparing MSKCC or IMDC risks between Responders and Non-Responders (two-sided Fisher's exact test); Kaplan–Meier curves of OS and PFS for patients with different MSKCC or IMDC risks (log-rank test). **b**, GSEA revealed the pathways associated with poor or intermediate MSKCC risk. **c**, GSEA revealed the pathways associated with poor or intermediate IMDC risk. **d**, Comparing Leibovich scores between Responders and Non-Responders (two-sided Fisher's exact test). **e**, Kaplan–Meier curves of OS and PFS for patients different Leibovich scores (log-rank test). Source data are provided as a Source data file.

# Supplementary Figure 5

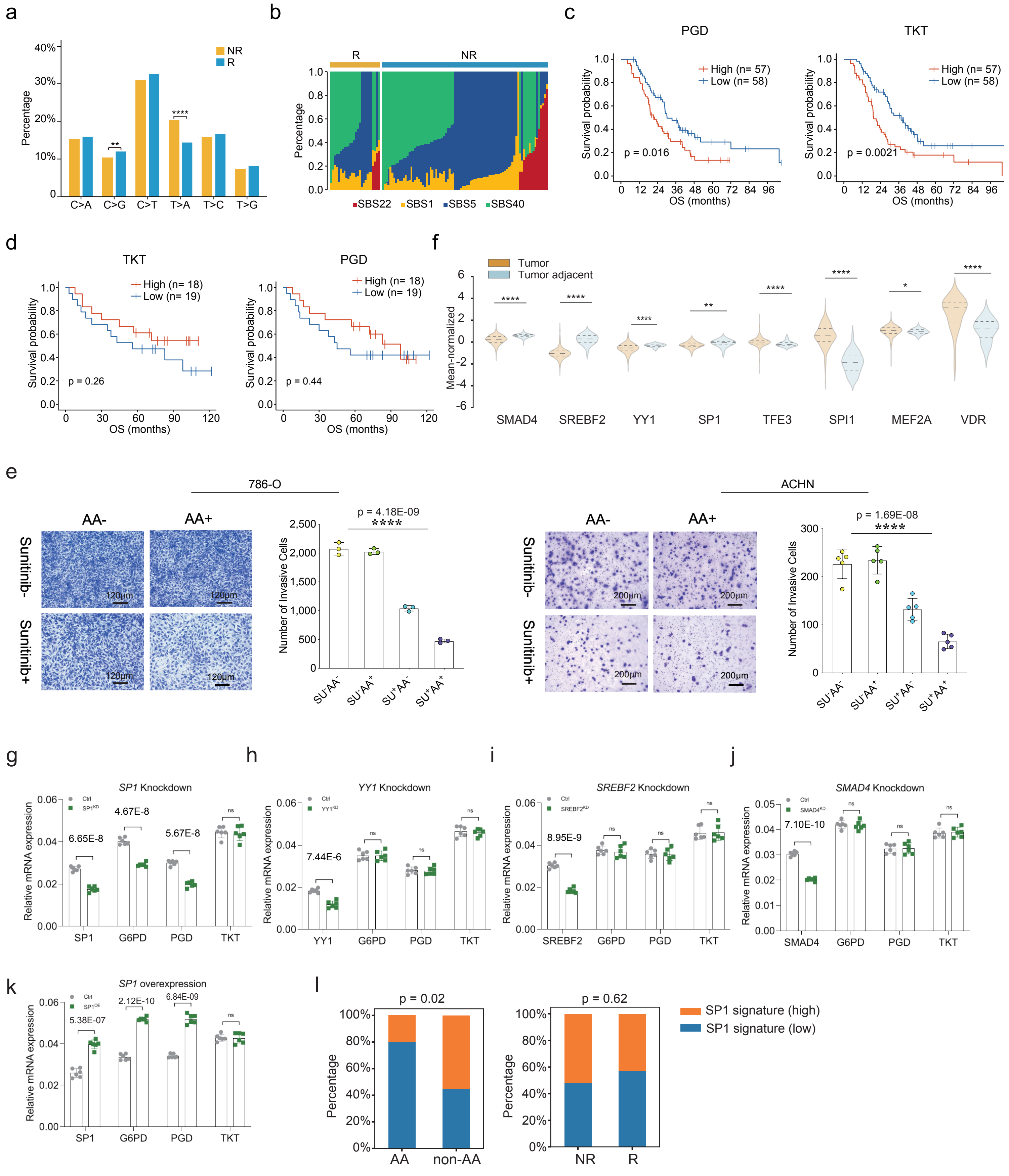

**Supplementary Figure 5. Profiles of Mutation Signatures and Their Effects on Proteome, and Clinical Outcomes.**

**a**, Frequency of substitution mutations in the Responders and Non-Responders (two-sided Fisher's exact test). **b**, Relative percentage of each mutation signature in the Responders and Non-Responders. **c**, Association between PGD and TKT protein levels and OS in Sunitinib treatment group. **d**, Association between PGD and TKT protein levels and OS in non-treated control group. **e**, Transwell detected the effect of AA and Sunitinib treatment on 786O (*n* = 3 independent experiments, data are presented as mean values +/- SD) and ACHN (*n* = 5 independent experiments, data are presented as mean values +/- SD) cells invasiveness (AA-100μM, Sunitinib-200nM). **f**. The expression level of eight TFs in between tumor tissue and tumor adjacent tissue in RCC cohort. **g-j**, The impacts of SP1, YY1, SREBF2, SMAD4 knockdown on PPP enzymes at mRNA level (*n* = 6 independent experiments, data are presented as mean values +/- SD, two-sided t test). **k**, SP1 overexpression upregulated PPP enzymes at mRNA level (*n* =6 independent experiments, data are presented as mean values +/- SD, two-sided t test). **l**, The stacked barplot depicting the distribution of SP1 signature (high) and SP1 signature (low) patients between AA, non-AA and Responder group and Non-Responder group. * $p < 0.05$, ** $p < 0.01$, ***$p < 1.0E-3$, **** $p < 1.0E-4$, ns. > 0.05. Source data are provided as a Source data file.

# Supplementary Figure 6

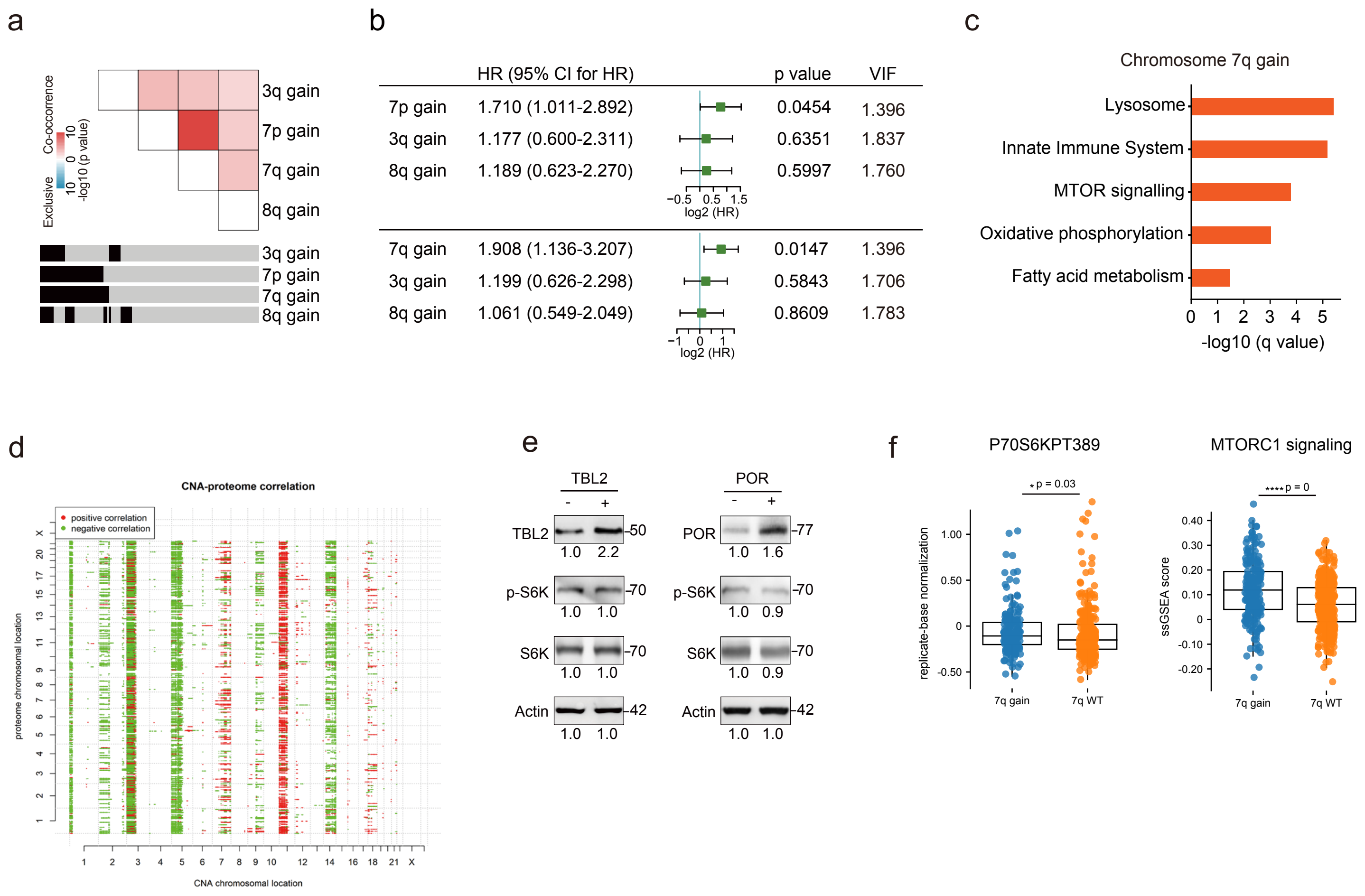

**Supplementary Figure 6. The Effects of CNA Events on Proteome, and Clinical Outcomes.**

**a,** Co-occurrence analysis of gains of 3q, 7p, 7q, and 8q (two-sided Fisher's exact test). **b**, Multivariate analysis of 3q gain, 7p/7q gain and 8q gain. Error bars indicates 95% confidence interval for HR (tumor samples, $n = 113$). **c**, Pathways enriched by 7q CN positively correlated proteins. **d**, Correlations of CNA (x axes) with protein abundance (y axes). Significant ($q < 0.10$) positive (red) and negative (green) correlations are shown. **e**, Effects of overexpression of TBL2 and POR on phosphorylation of S6K. Numerical values below the gels indicate quantification of the bands relative to control. **f**, Comparison of pS6K and mTORC1 signaling scores between 7q gain ccRCC ($n$ = 174) and 7q WT ccRCC patients ($n$ = 272) in the TCGA RPPA and transcriptome data (two-sided Wilcoxon rank-sum test). Boxplots show the median (central line), the 25–75% interquartile range (IQR) (box limits), the ±1.5×IQR (whiskers). Source data are provided as a Source data file.

# Supplementary Figure 7

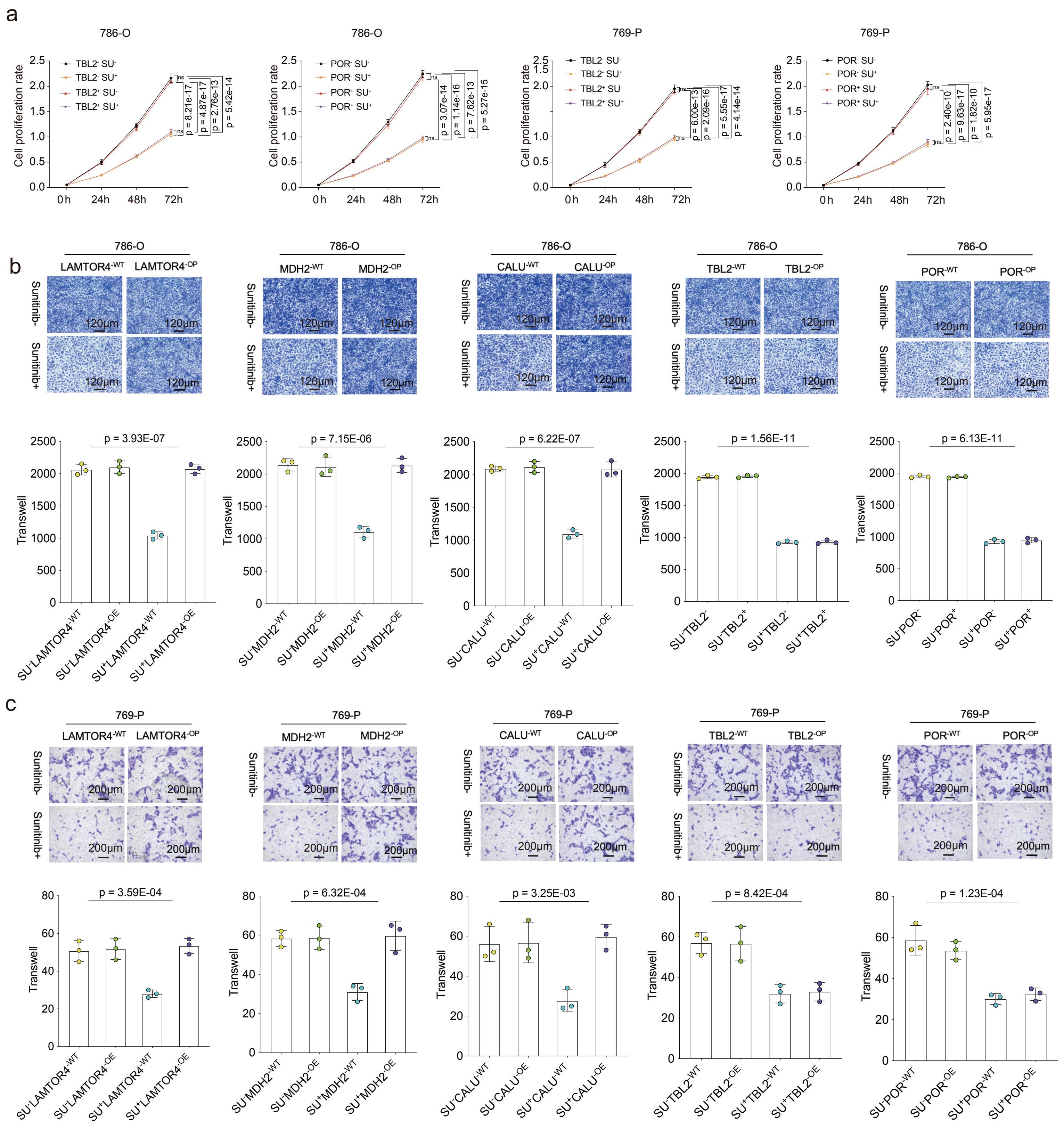

**Supplementary Figure 7. The experiment evidence for the effects of CNA Events on Proteome, and Clinical Outcomes.**

**a,** CCK-8 detected the effect of TBL2 and POR overexpression and Sunitinib treatment on cell proliferation, in 786-O and 769-P cell lines, respectively (Sunitinib-200nM, *n* = 9 independent experiments, data are presented as mean values +/- SD, two-sided t test). **b-c**, Up, Transwell detected the effect of LAMTOR4, MDH2, CALU, TBL2 and POR overexpression and Sunitinib treatment on cell invasiveness in 786-O and 769-P cells (*n* = 3 independent experiments, data are presented as mean values +/- SD), respectively. Down, Quantification of transwell results. * $p < 0.05$, ** $p < 0.01$, ***$p < 1.0E-3$, **** $p < 1.0E-4$, ns. > 0.05. Source data are provided as a Source data file.

# Supplementary Figure 8

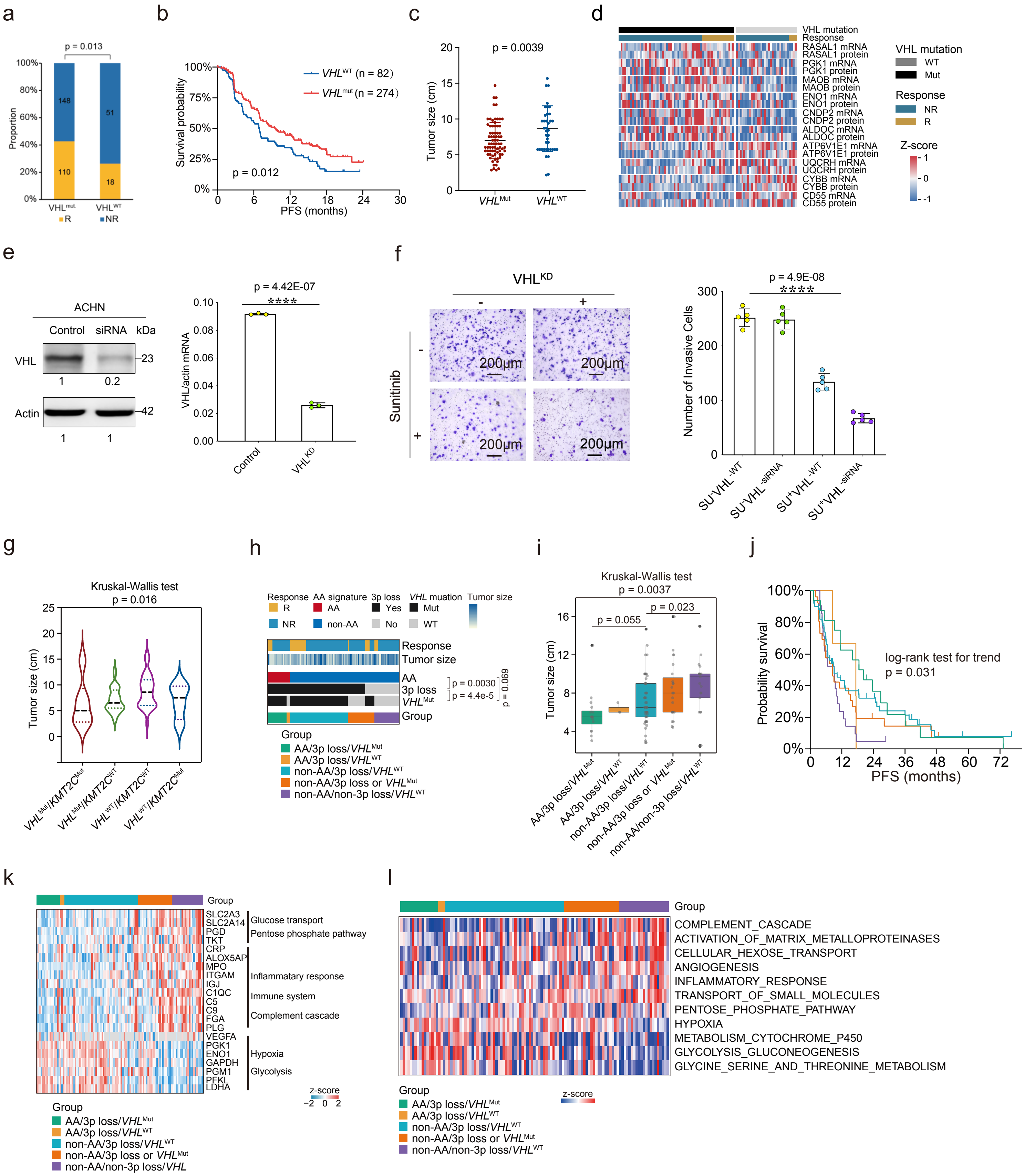

**Supplementary Figure 8. Genomic Alteration Groups Associated Proteomic and Clinical Features.**

**a**, Stacked graph bars represent the number of responses for patients treated with Sunitinib in IMmotion151 study; complete response (CR), partial response (PR), stable disease (SD), progression disease (PD) or not evaluated (NE). P value is derived from two-sided Fisher's exact test. **b**, Kaplan–Meier curves of progression-free survival (PFS) for patients with or without *VHL* mutation in IMmotion151 study. **c**, Comparisons of tumor size between patients with ($n$ = 74) or without ($n$ = 39) *VHL* mutation (two-sided t test). Data are shown as mean ± SD. **d**, Differentially level of RNAs and proteins between VHL mutation and WT groups. The values were transformed by z-score. **e**, Transfection efficiency of *VHL* siRNA detected by qRT-PCR ($n$ = 3 independent experiments, data are presented as mean values +/- SD, **** p <1.0E-4) and western blot. Numerical values below the gels indicate quantification of the bands relative to control. **f**, left panel, Transwell detected the effect of VHL knockdown and Sunitinib treatment on cell invasiveness in ACHN cell. Right panel, Quantification of transwell results ($n$ = 5 independent experiments, data are presented as mean values +/- SD, **** p <1.0E-4). **g,** Violin plot showing tumor sizes among different *VHL* and *KMT2C* genotypes (Kruskal−Wallis test). **h**, Comprehensive analysis of mutation signatures, CNAs, and gene mutations revealed the co-occurrence of AA signature, 3p loss, and *VHL* mutations. This cohort was divided into five groups based on their alteration status (Group1: AA/3p loss/$VHL^{Mut}$; Group2: AA/3p loss/$VHL^{WT}$; Group3: non-AA/3p loss/$VHL^{WT}$; Group4: non-AA/3p loss or $VHL^{Mut}$; Group5: non-AA/non-3p loss/$VHL^{WT}$). p, The difference of tumor size in five groups with different genomic alteration status. **i**, Boxplot showing the difference of tumor size in five groups with different genomic alteration status (Kruskal−Wallis test). Boxplots show the median (central line), the 25–75% interquartile range (IQR) (box limits), the ±1.5×IQR (whiskers). **j**, Kaplan-Meier curves of PFS for five genomic subgroups (log-rank test for trend). **k**, Differentially expressed proteins in the five groups and their associated biological pathways. The values were transformed by z-score. **l**, Heatmap of pathway

ssGSEA scores in the five genomic alteration groups. Source data are provided as a Source data file.

# Supplementary Figure 9

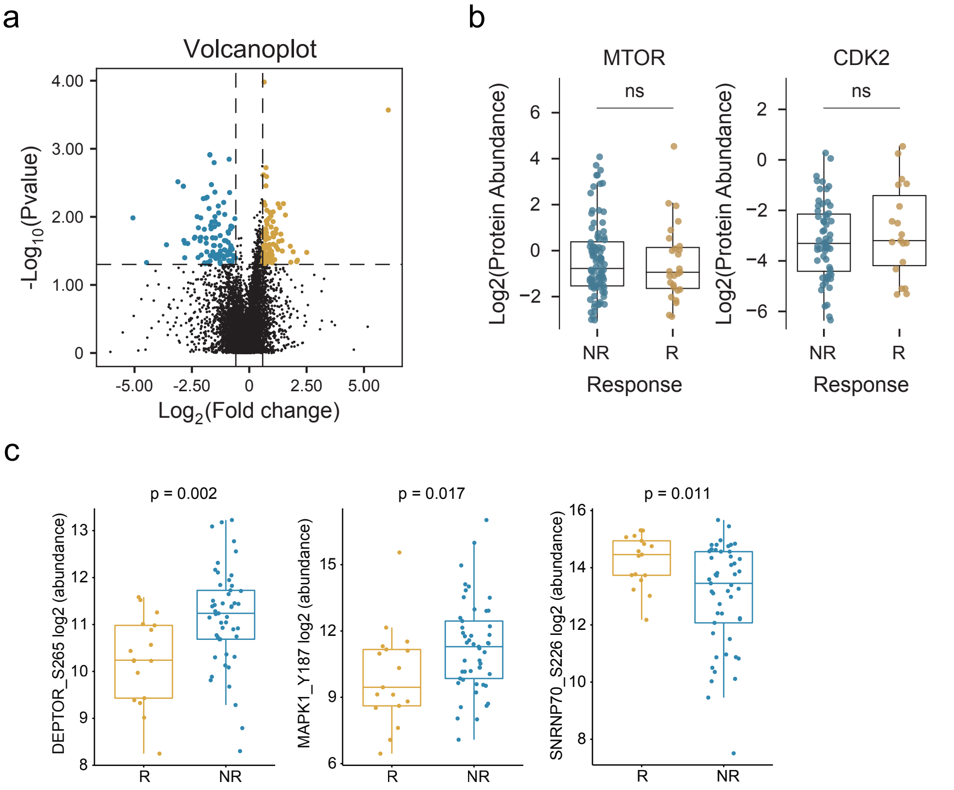

**Supplementary Figure 9. Sunitinib Therapeutic Outcomes Associated with Proteomic and Clinical Features**

**a**, Volcano plot showing the differentially expressed proteins (DEPs) ($p$ value $< 0.05$, FC $> 1.5$) between Responders and Non-Responders. **b**, The distribution of MTOR and CDK2 protein abundance between Responders ($n = 21$) and Non-Responders ($n = 73$). P-values were derived by the two-sided ranksums test. Boxplots show the median (central line), the 25–75% interquartile range (IQR) (box limits), the ±1.5×IQR (whiskers). **c**, Abundances of phosphosites (DEPTOR pS265, MAPK1 pY187, SNRNP70 pS226) between Responders ($n = 17$) and Non-Responders ($n = 49$) (two-sided Wilcoxon rank-sum test). Boxplots show the median (central line), the 25–75% interquartile range (IQR) (box limits), the ±1.5×IQR (whiskers). Source data are provided as a Source data file.

# Supplementary Figure 10

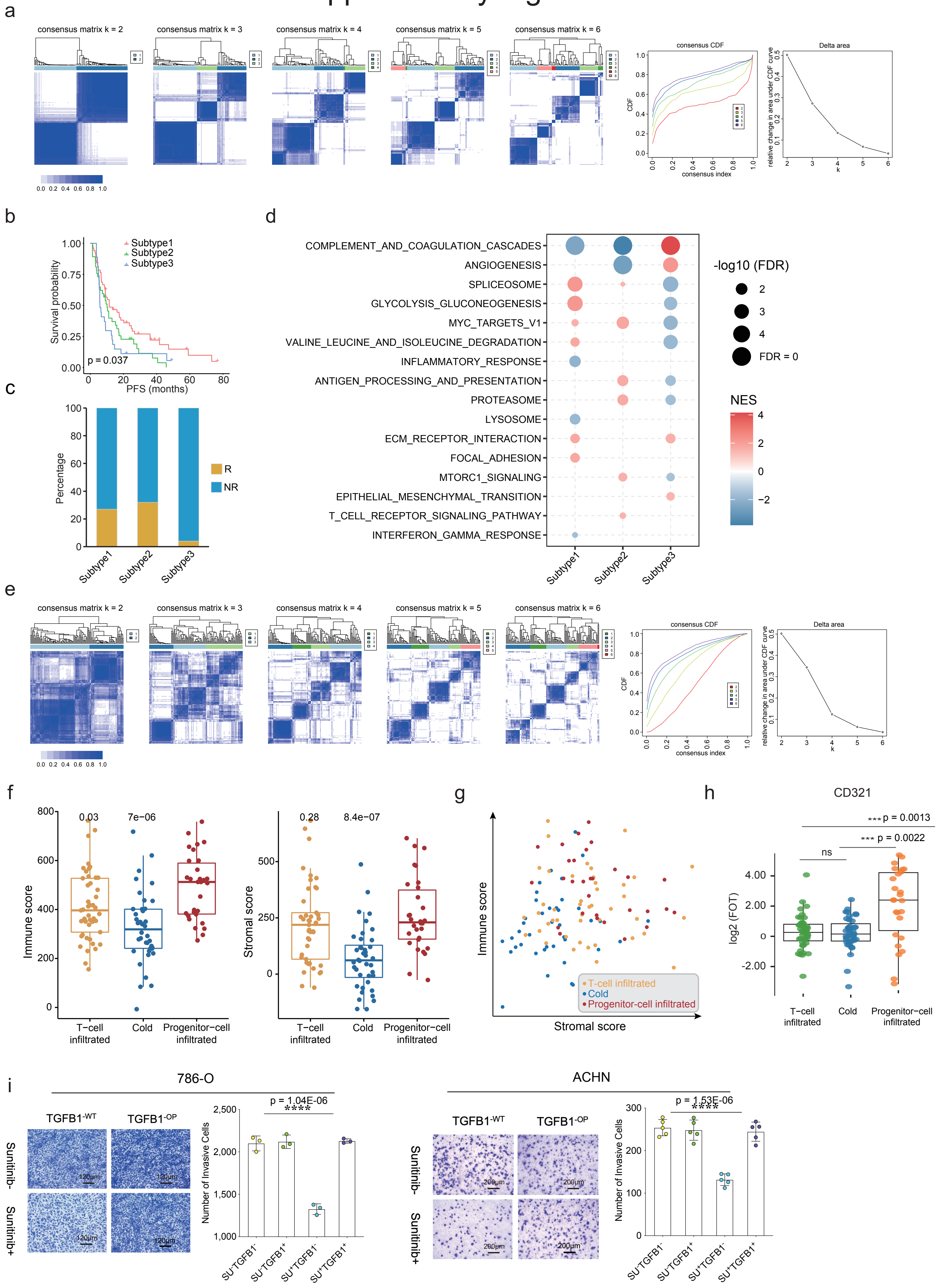

**Supplementary Figure 10. Heterogeneity and Immune infiltrations in Responders and Non-Responders**

**a**, Consensus matrices of the 115 ccRCC samples from k = 2 to k = 6; Cumulative distribution function plot, and delta plot corresponding to the consensus matrices from k = 2 to k = 6. **b**, Kaplan–Meier curves of PFS for the three proteomic subtypes (log-rank test).**c**, Proportions of Responders and Non-Responders among the three proteomic subtypes. **d**, GSEA of the three proteomic subtypes. **e**, Consensus matrices of the 115 ccRCC xCell inferred TME components from k = 2 to k = 6; Cumulative distribution function plot, and delta plot corresponding to the consensus matrices from k = 2 to k = 6. **f-g**, The stromal scores and immune scores of three immune subtypes (T-cell infiltrated cluster, $n = 51$; Cold cluster, $n = 37$; Progenitor-cell infiltrated cluster, $n = 27$, two-sided Kruskal−Wallis test). Boxplots show the median (central line), the 25–75% interquartile range (IQR) (box limits), the ±1.5×IQR (whiskers). **h**, Abundance of CD321 between three immune subtypes (Wilcoxon rank-sun test). Boxplots show the median (central line), the 25–75% interquartile range (IQR) (box limits), the ±1.5×IQR (whiskers). **i**, left panel, Transwell detected the effect of TGFB1 overexpression and Sunitinib treatment on cell invasiveness in 786-O ($n = 3$ independent experiments, data are presented as mean values +/- SD, **** p <1.0E-4) and ACHN ($n = 5$ independent experiments, data are presented as mean values +/- SD, **** p <1.0E-4) cells, respectively. Right panel, Quantification of transwell results in 786-O and ACHN cells, respectively. Source data are provided as a Source data file.

# Supplementary Figure 11

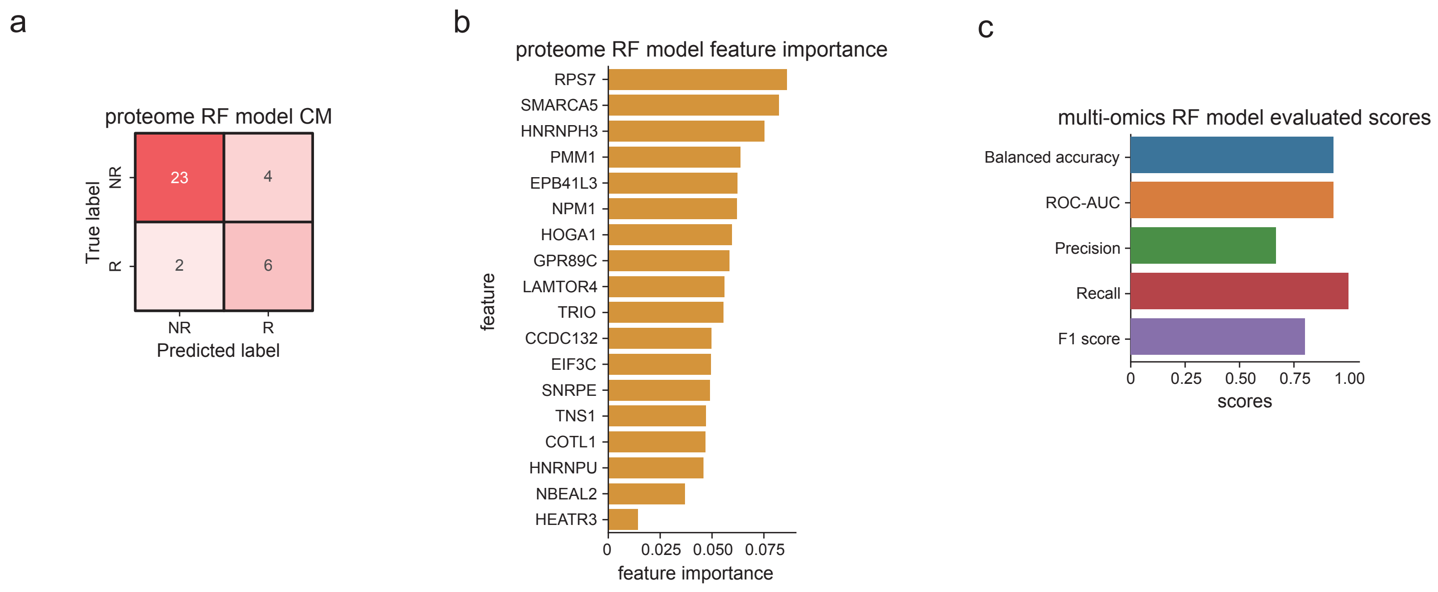

**Supplementary Figure 11. Proteomic Classifier to Predict Sunitinib Response**

**a**, The confusion matrix of test cohort for proteome-based RF. **b**, The feature importance of proteome-based RL model. **c**, Bar plot depicting the different evaluation score of multi-omics-base RF model including balanced accuracy, ROC AUC, precision, recall, and F1 score. Source data are provided as a Source data file.

# Supplementary Figure 12

a

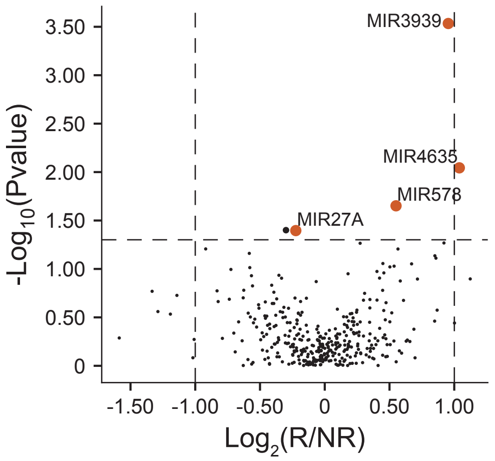

**Supplementary Figure 12. miRNAs dysregulation associated with the response to Sunitinib.**

**a,** Scatter plot showing the overrepresented miRNAs which regulated the differential expressed proteins of Responders and Non-Responders.
