# Supplementary material for: Proteogenomics of clear cell renal cell carcinoma response to tyrosine kinase inhibitor
Source: Nat Commun. 2023 Jul 17;14:4274. doi: 10.1038/s41467-023-39981-6 (PMC10352361; doi:10.1038/s41467-023-39981-6)
Supplement: Supplementary file 3 — Description of Additional Supplementary Files [file 41467_2023_39981_MOESM3_ESM.pdf]

Description of Additional Supplementary Files

Supplementary Data 1: Clinical characteristics of 115 Chinese ccRCC patients undergoing Sunitinib treatment.

Supplementary Data 2: Genomic Alterations in 115 ccRCC samples.

Supplementary Data 3: Significantly differentially altered in the Responder and Non Responder groups.

Supplementary Data 4: Expression matrix of 115 pairs of Chinese ccRCC samples.

Supplementary Data 5: Differential Analysis between Sunitinib Therapeutic Responders and Non-Responders at Proteome and Phosphoproteome Levels.

Supplementary Data 6: Immune Infiltration Analysis of 115 ccRCC tumor samples.
