# Supplementary material for: Proteogenomics of clear cell renal cell carcinoma response to tyrosine kinase inhibitor
Source: Nat Commun. 2023 Jul 17;14:4274. doi: 10.1038/s41467-023-39981-6 (PMC10352361; doi:10.1038/s41467-023-39981-6)
Supplement: Supplementary file 10 — Reporting Summary [file 41467_2023_39981_MOESM10_ESM.pdf]

nature portfolio

Corresponding author(s): Chen Ding, Dingwei Ye, Yuanyuan Qu, Jian-Yuan Zhao

Last updated by author(s): Jun 15, 2023

Reporting Summary

Nature Portfolio wishes to improve the reproducibility of the work that we publish. This form provides structure for consistency and transparency in reporting. For further information on Nature Portfolio policies, see our Editorial Policies and the Editorial Policy Checklist.

Statistics

For all statistical analyses, confirm that the following items are present in the figure legend, table legend, main text, or Methods section.

n/a	Confirmed	
☐	☒	The exact sample size (*n*) for each experimental group/condition, given as a discrete number and unit of measurement
☐	☒	A statement on whether measurements were taken from distinct samples or whether the same sample was measured repeatedly
☐	☒	The statistical test(s) used AND whether they are one- or two-sided<br>*Only common tests should be described solely by name; describe more complex techniques in the Methods section.*
☐	☒	A description of all covariates tested
☐	☒	A description of any assumptions or corrections, such as tests of normality and adjustment for multiple comparisons
☐	☒	A full description of the statistical parameters including central tendency (e.g. means) or other basic estimates (e.g. regression coefficient) AND variation (e.g. standard deviation) or associated estimates of uncertainty (e.g. confidence intervals)
☐	☒	For null hypothesis testing, the test statistic (e.g. *F*, *t*, *r*) with confidence intervals, effect sizes, degrees of freedom and *P* value noted<br>*Give P values as exact values whenever suitable.*
☒	☐	For Bayesian analysis, information on the choice of priors and Markov chain Monte Carlo settings
☒	☐	For hierarchical and complex designs, identification of the appropriate level for tests and full reporting of outcomes
☐	☒	Estimates of effect sizes (e.g. Cohen's *d*, Pearson's *r*), indicating how they were calculated

Our web collection on statistics for biologists contains articles on many of the points above.

Software and code

Policy information about availability of computer code

Data collection	Samples were analysed on a Q Exactive HF-X mass spectrometer (Thermo Fisher Scientific, Rockford, IL, USA) coupled with high-performance liquid chromatography (EASY-nLC 1200 System, Thermo Fisher Scientific).The mass spectrometry data were acquired using the Xcalibur software v2.2 (Thermo Fischer Scientific). Whole exome sequencing (WES) was all performed on the Nextseq CN500 platform (Illumina). RNA sequencing was performed on an Illumina HiSeq 4000 platform.

Data analysis

The mass spectrometry raw files were processed in Firmiana, searched against human National Center for Biotechnology Information (NCBI) RefSeq protein database (updated on 04-07-2013, 32,015 entries) using Mascot 2.4 (Matrix Science Inc, London, UK). Statistical analyses were realized by R (v3.6.0). Approaches or algorithms used for the proteome data annotation include ConsensusClusterPlus (v1.52.0), factoextra (v1.0.6), survival (v3.2-3), survminer (v0.4.8), pheatmap (v1.0.12), ESTIMATE (v1.0.11), GSEA software (v4.0.3), ssGSEA (v2.0), GSVA (v1.34.0), corrplot (v0.84), DoRothEA (v1.6.0). The mapped reads were assembled into transcripts or genes by using StringTie software (v2.1.4). Gene annotation was performed using online tools DAVID and ConsensusPathDB. Whole exome sequencing data was analyzed using GATK (v3.8.1.0 & v4.1.2.0), Maftools (v3.10), SigProfilerExtractor (v1.1), GISTIC2.0 and multiOmicsViz (v1.10.0). Classifier construction was analyzed using Python (version 3.9.0) using the following libraries: scikit-learn (version 1.2.1), numpy (version 1.16.4), scipy (version 1.3), pandas (version 1.5.2). Standard statistical tests were used to analyze the clinical data, including but not limited to Student's t test, Wilcoxon test, Fisher's exact test, Kruskal-Wallis test, Spearman's correlation test, Pearson's correlation test, log-rank test. Unless otherwise specified, all statistical tests were two-sided, and statistical significance was considered when p value < 0.05. Kaplan–Meier plots (log-rank test) were used to describe overall survival. Variables associated with overall survival were identified using univariate Cox proportional hazards regression models. All the analyses of clinical data were performed in R and GraphPad Prism. For functional experiments, each was repeated at least three times independently, and results were expressed as mean ± standard deviation (SD). Statistical analysis was performed using GraphPad Prism.

For manuscripts utilizing custom algorithms or software that are central to the research but not yet described in published literature, software must be made available to editors and reviewers. We strongly encourage code deposition in a community repository (e.g. GitHub). See the Nature Portfolio guidelines for submitting code & software for further information.

Data

Policy information about availability of data

All manuscripts must include a data availability statement. This statement should provide the following information, where applicable:

- Accession codes, unique identifiers, or web links for publicly available datasets
- A description of any restrictions on data availability
- For clinical datasets or third party data, please ensure that the statement adheres to our policy

The proteome and phosphoproteome raw datasets have been deposited to the ProteomeXchange Consortium (dataset identifier: PXD042844) via the iProX partner repository (https://www.iprox.cn/) under Project ID: IPX0002932000. The raw WES and RNA data are available in the Genome Sequence Archive (GSA) under restricted access HRA003490. The raw sequencing data are available under controlled access due to data privacy laws related to patient consent for data sharing and the data should be used for research purposes only. According to the guidelines of GSA-human, all non-profit researchers are allowed access to the data, and the Principle Investigator of any research group can apply for Controlled-access of the data. The user can register and log in to the GSA database website (https://ngdc.cncb.ac.cn/gsa-human/) and follow the guidance of "Request Data" to request the data step by step. The approximate response time for accession requests is about 2 weeks. The access authority can be obtained for Research Use Only. The user can also contact the corresponding author directly. Once access has been granted, the data will be available to download for 3 months. The remaining data are available within the Article, Supplementary Information, or Source Data file. Source data are provided with this paper.

Research involving human participants, their data, or biological material

Policy information about studies with human participants or human data. See also policy information about sex, gender (identity/presentation), and sexual orientation and race, ethnicity and racism.

Reporting on sex and gender

There were 115 people in the cohort, 91 males and 24 females.

Reporting on race, ethnicity, or other socially relevant groupings

This study included 115 Chinese patients with advanced ccRCC, who were treated with TKIs at the Department of Urology of Fudan University Shanghai Cancer Center (FUSCC, Shanghai, China) from Jan 2008 to Dec 2019.

Population characteristics

This study included 115 patients with advanced ccRCC, who were treated with TKIs at the Department of Urology of Fudan University Shanghai Cancer Center (FUSCC, Shanghai, China) from Jan 2008 to Dec 2019, with an age range 21–84. All electronic medical records were screened retrospectively. Among the 115 cases, 47 cases were localized ccRCC at the time of surgery and were included due to the subsequent development of metastatic disease. The median follow-up was 28.4 months (range, 4.2–127.5 months). At the last follow-up, 102 patients (88.7%) had progressive disease and 84 patients (73.0%) had died of ccRCC.

Recruitment

We screened consecutive patients who underwent radical or partial nephrectomy for the treatment of renal tumors at the Department of Urology of Fudan University Shanghai Cancer Center (FUSCC, Shanghai, China) from Jan 2008 to Dec 2019. All patients were treatment-naïve before surgery and received Sunitinib treatment after surgery. In total, 68 advanced and 47 recurrent tumors were included in these study.

Ethics oversight

The study was compliant with the ethical standards of Helsinki Declaration II and was approved by the institutional review board of Fudan University Shanghai Cancer Center (FUSCC) (050432-4-1911D). Written informed consent was obtained from each patient before any study-specific investigation was conducted.

Note that full information on the approval of the study protocol must also be provided in the manuscript.

Field-specific reporting

Please select the one below that is the best fit for your research. If you are not sure, read the appropriate sections before making your selection.

☒ Life sciences ☐ Behavioural & social sciences ☐ Ecological, evolutionary & environmental sciences

For a reference copy of the document with all sections, see nature.com/documents/nr-reporting-summary-flat.pdf

Life sciences study design

All studies must disclose on these points even when the disclosure is negative.

Sample size	We retrospectively collected paired tumor and tumor-adjacent ccRCC samples based on strict criteria from 115 Chinese patients treated with Sunitinib, comprising 68 advanced and 47 recurrent tumors for proteogenomic analysis. The 47 recurrent cases were localized ccRCC at the time of surgery and were enrolled due to the subsequent development of metastatic disease. No statistical method was used to predetermine sample size. The functional and biological experiments were performed with at least three biological replicates to allow statistical significance testing. The sample size of ccRCC patients was based on published paper in the ccRCC field (PMID: 37074911).
Data exclusions	Whole exome sequencing was performed using 113 paired samples, except for 2 patients due to low DNA quality.
Replication	The functional and biological experiments were performed with at least three biological replicates to allow statistical significance testing.
Randomization	For multi-omic analysis, samples of ccRCC patients were randomly divided into groups to avoid bias for protein/phosphoprotein, RNAs quantification. For the classifier, samples were randomly split into training set and test set.
Blinding	The investigators who measured protein expression/mRNA expression, WES data were blinded to patient information. The investigators who performed IHC were blinded to clinical information of ccRcc patients. For sample processing, PCA, consensus clustering analysis, all investigators were blinded to clinical information (including TNM stage, ISUP grade, and patients outcomes).

Reporting for specific materials, systems and methods

We require information from authors about some types of materials, experimental systems and methods used in many studies. Here, indicate whether each material, system or method listed is relevant to your study. If you are not sure if a list item applies to your research, read the appropriate section before selecting a response.

Materials & experimental systems

n/a	Involved in the study	
☐	☒	Antibodies
☐	☒	Eukaryotic cell lines
☒	☐	Palaeontology and archaeology
☒	☐	Animals and other organisms
☒	☐	Clinical data
☒	☐	Dual use research of concern
☒	☐	Plants

Methods

n/a	Involved in the study	
☒	☐	ChIP-seq
☒	☐	Flow cytometry
☒	☐	MRI-based neuroimaging

Antibodies

Antibodies used	1, Anti-G6PD antibody (Proteintech, #25413-1-AP, 1:1000 dilution)<br>2, Anti-PGD antibody (Proteintech, #14718-1-AP, 1:1000 dilution)<br>3, Anti-TKT antibody (Invitrogen, #PA5-54301, 1:1000 dilution)<br>4, Anti-LAMTOR4 antibody (Invitrogen, #PA5-54301, 1:1000 dilution)<br>5, Anti-MDH2 antibody (Abclonal, #A13516, 1:1000 dilution)<br>6, Anti-CALU antibody (Abclonal, #A6538, 1:1000 dilution)<br>7, Anti-S6K antibody (Cell Signaling Technology, #5707, 1:1000 dilution)<br>8, Anti-pS6K antibody (Cell Signaling Technology, #9209, 1:1000 dilution)<br>9, Anti-VHL antibody (Abcam, #ab270968, 1:5000 dilution)<br>10, Anti-Actin antibody (Genscript, #A00702, 1:800 dilution)
Validation	1, Anti-G6PD antibody: https://www.ptglab.com/products/pictures/pdf/25413-1-AP.pdf<br>2, Anti-PGD antibody: https://www.ptglab.com/products/pictures/pdf/14718-1-AP.pdf<br>3, Anti-TKT antibody: https://www.thermofisher.cn/order/genome-database/dataSheetPdf?producttype=antibody&productsubtype=antibody_primary&productId= PA5-56165&version=216<br>4, Anti-LAMTOR4 antibody: https://www.thermofisher.cn/order/genome-database/dataSheetPdf?producttype=antibody&productsubtype=antibody_primary&productId=PA5-54301&version=216<br>5, Anti-MDH2 antibody: https://abclonal.com.cn/Datasheet/Antibodies/A13516.pdf

6, Anti-CALU antibody: https://abclonal.com.cn/Datasheet/Antibodies/A6538.pdf
7, Anti-S6K antibody: https://media.cellsignal.com/pdf/5707.pdf
8, Anti-pS6K antibody: https://media.cellsignal.com/pdf/9209.pdf
9, Anti-VHL antibody: https://www.abcam.cn/nnmt-antibody- ab270968.pdf
10, Anti-Actin antibody: https://www.genscript.com/product/documents/down?doc_name=A00702-200_2016-08-17.pdf&file=scm_files/productFile_notes/2016/08/17/A00702-200_US_2016-08-17.pdf

Eukaryotic cell lines

Policy information about cell lines and Sex and Gender in Research

Cell line source(s)	ACHN (Cat#HTB-44; RRID: CVCL_1067), 786-O cells (Cat# CRL-1932; RRID: CVCL_1051), 769-P cells (CRL-1933; RRID: CVCL_1050) were obtained from ATCC.
Authentication	All cell lines were validated by Short tandem repeat (STR) profiling at ATCC Facility.
Mycoplasma contamination	All cell lines were tested negtaive for mycoplasma contamination.
Commonly misidentified lines (See ICLAC register)	No commonly misidentified cell lines were used.
